# Secondary Prevention in Hereditary Breast and/or Ovarian Cancer Syndromes Other Than BRCA

**DOI:** 10.1155/2020/6384190

**Published:** 2020-07-14

**Authors:** Claudia Piombino, Laura Cortesi, Matteo Lambertini, Kevin Punie, Giovanni Grandi, Angela Toss

**Affiliations:** ^1^Department of Oncology and Hematology, University Hospital of Modena, Modena, Italy; ^2^Department of Medical Oncology, U.O.C Clinica di Oncologia Medica, IRCCS Ospedale Policlinico San Martino, Genova, Italy; ^3^Department of Internal Medicine and Medical Specialties (DiMI), School of Medicine, University of Genova, Genova, Italy; ^4^Department of General Medical Oncology, Multidisciplinary Breast Center, Leuven Kanker Instituut, University Hospitals Leuven, Leuven, Belgium; ^5^Department of Obstetrics and Ginecology, University Hospital of Modena, Modena, Italy; ^6^Department of Surgery, Medicine, Dentistry and Morphological Sciences with Transplant Surgery, Oncology and Regenerative Medicine Relevance, University of Modena and Reggio Emilia, Modena, Italy

## Abstract

BRCA1- and BRCA2-associated hereditary breast and ovarian cancer syndromes are among the best-known and most extensively studied hereditary cancer syndromes. Nevertheless, many patients who proved negative at BRCA genetic testing bring pathogenic mutations in other suppressor genes and oncogenes associated with hereditary breast and/or ovarian cancers. These genes include *TP53* in Li–Fraumeni syndrome, *PTEN* in Cowden syndrome, mismatch repair (*MMR*) genes in Lynch syndrome, *CDH1* in diffuse gastric cancer syndrome, *STK11* in Peutz–Jeghers syndrome, and *NF1* in neurofibromatosis type 1 syndrome. To these, several other genes can be added that act jointly with *BRCA1* and *BRCA2* in the double-strand break repair system, such as *PALB2*, *ATM*, *CHEK2*, *NBN*, *BRIP1*, *RAD51C,* and *RAD51D*. Management of primary and secondary cancer prevention in these hereditary cancer syndromes is crucial. In particular, secondary prevention by screening aims to discover precancerous lesions or cancers at their initial stages because early detection could allow for effective treatment and a full recovery. The present review aims to summarize the available literature and suggest proper screening strategies for hereditary breast and/or ovarian cancer syndromes other than BRCA.

## 1. Introduction

**Figure 1:**
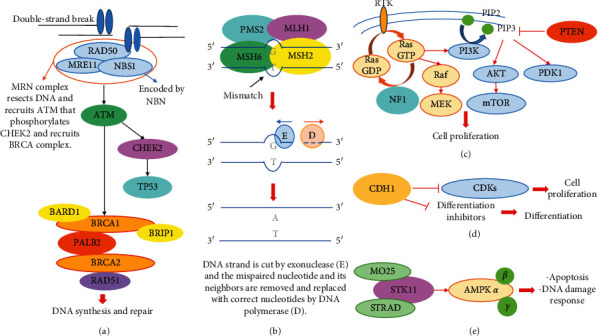
Molecular pathways involved in hereditary cancer risk. Susceptibility genes described in the text are reported in bold. G: guanine, T: thymine, A: adenine, E: exonuclease, D: DNA polymerase, RTK: receptor tyrosine kinase, PIP2: phosphatidylinositol 4,5-bisphosphate, PIP3: phosphatidylinositol (3,4,5)-trisphosphate, GDP: guanosine diphosphate, GTP: guanosine triphosphate, CDKs; cyclin-dependent kinases, and AMPK: 5′ adenosine monophosphate-activated protein kinase. (a) Homologus recombination (HR), (b) mismatch repair (MMR), (c) PTEN and NF1 pathways, (d) CDH1 pathway, and (e) STK11 pathway.

In hereditary cancer syndromes (HCSs), inherited mutations lead to an increased risk of developing certain tumors, frequently at an earlier age than in the rest of the population [1]. Elevated cancer risk is usually due to a mutation in a single gene involved in cell cycle regulation or in DNA damage repair mechanisms (Figure 1). The most widely known HCSs include hereditary breast and ovarian cancer syndromes due to mutations in the *BRCA1*/*2* genes [2, 3], Li–Fraumeni syndrome due to mutations in *TP53* [4], Cowden syndrome due to mutations in *PTEN* [5], Lynch syndrome, in which mutations in the DNA mismatch repair system are involved [6, 7], diffuse gastric cancer syndrome caused by *CDH1* gene mutation [8], Peutz–Jeghers syndrome caused by mutations in the *STK11* [9] gene, and neurofibromatosis type 1 syndrome caused by *NF1* mutations [10]. Additionally, pathogenic alterations in *PALB2* [11], *ATM* [12], *CHEK2* [13], and *NBN* [14] are correlated with an increased risk for breast cancer and/or other cancers, whereas other genes such as *BRIP1*, *RAD51C,* and *RAD51D* are associated with an increased ovarian cancer risk [15].

Management of cancer prevention is crucial in HCSs. Cancer prevention can be divided into primary and secondary strategies [16–23]. The aim of the primary prevention is to avoid cancer development by strategies including health counselling and education, environmental controls, prophylactic surgery, and chemoprevention. Secondary prevention by screening aims to discover precancerous lesions or cancers at their initial stages because early detection could allow for an effective treatment and full recovery. Strategies of primary and secondary cancer prevention are well established in the setting of *BRCA*-associated breast and ovarian cancer. For all other syndromes, on the other hand, the most appropriate screening protocol is still debated.

This review aims to summarize the available literature and suggest proper screening strategies for hereditary breast and/or ovarian cancer syndromes other than those associated with *BRCA* mutations.

## 2. Li–Fraumeni Syndrome

Li–Fraumeni syndrome is a rare autosomal dominant cancer predisposition syndrome that involves a germline mutation of the tumor protein 53 (*TP53* gene) [4]. The estimated prevalence of pathogenetic germline *TP53* mutations ranges from 1/10,000 to 1/25,000 in the UK and is estimated at 1/20,000 in the US [24]. The lifetime cancer risk in individuals with Li–Fraumeni syndrome is ≥70% for men and ≥90% for women [25]. Five cancer types account for the majority of Li–Fraumeni tumors: adrenocortical carcinomas, breast cancer, central nervous system tumors, osteosarcomas, and soft-tissue sarcomas [26]. Individuals with Li–Fraumeni syndrome are also at an increased risk of developing hematologic tumors (leukaemia and lymphomas), gastrointestinal cancers, gynecological tumors, and melanoma [4].

Surveillance recommendations for individuals with Li–Fraumeni syndrome are primarily based on the “Toronto protocol” [27]. For breast cancer, screening recommendations advise starting with clinical breast examination once in every 6–12 months from the age of 20. Annual breast MRI screening with contrast is suggested from 20 to 75 years of age. Given the increased sensitivity to ionizing radiation and the increased risk for radiation-induced malignancies in patients with germline pathogenic *TP53* variants, there are concerns about the safety of repeated mammograms. There is no consensus in the literature, but in light of the limited additional sensitivity of mammography when MRI and alternating whole-body diffusion-weighted MRI are used, risks seem to outweigh benefits [28–30]. In case of family history of breast cancer diagnosed earlier than 20 years of age, breast MRI might start five years prior to the earliest age of diagnosis. Although there are no data regarding risk-reduction surgery in women with Li–Fraumeni syndrome, the option of risk-reducing bilateral mastectomy should be considered and discussed with female patients [6, 28]. Concerning gastrointestinal cancer, colonoscopy and upper endoscopy should be performed once in every 2–5 years starting from 25 years of age or five years prior to the earliest case of colorectal cancer in the family. Moreover, annual dermatologic examination is recommended from 18 years of age due to increased skin cancer risk, although less well-defined.

As regards many of the other cancers associated with Li–Fraumeni syndrome, early symptom-based detection is quite difficult. General recommendations include complete physical examination (including blood pressure evaluation, full neurologic exams, assessment of growth, sudden weight gain or loss, Cushingoid appearance, or signs of virilization in children) once in every 3–4 months until the age of 18 and then once in every six months. Annual whole-body diffusion-weighted MRI could allow for early detection of adrenocortical carcinomas and sarcomas, based on the results of multiple international trials [27, 31, 32]. As far as the central nervous system is concerned, the Toronto protocol with modifications [27] recommends annual brain MRI: first, MRI with contrast and then without contrast if previous MRI is normal and no new abnormality has been detected, in order to minimize the potential for gadolinium accumulation in the basal ganglia in individuals undergoing multiple enhanced MRIs [28]. Periodic blood tests can be considered in those at increased risk for myelodysplastic syndrome or leukaemia due to prior cancer treatments [28].

## 3. Cowden Syndrome

Cowden syndrome is the most prevalent PTEN hamartoma tumor syndrome associated with multiple hamartomatous and/or cancerous lesions in the skin, mucous membranes, thyroid, breast, endometrium, kidney, and brain [33]. Affected individuals usually have macrocephaly, trichilemmomas, and papillomatous papules, and the syndrome becomes apparent by the late 20s [5]. The estimated incidence of Cowden syndrome is 1/200,000, but it is likely to be underestimated due to the difficulties of making a clinical diagnosis of the disease [34]. Cowden syndrome is an autosomal dominant disorder due to germline *PTEN* mutation in 80% of cases [35].

The lifetime risk of developing breast cancer is 85%, with an average age at diagnosis between 38 and 46 years [5]. NCCN guidelines [6] recommend clinical breast examination once in every six months beginning at 25 years of age and annual mammogram and breast MRI screening with contrast starting at 30–35 years of age. However, screening should start 5–10 years prior to the earliest case of breast cancer in the family. Although there are no data regarding risk-reduction surgery in women with Cowden syndrome, the option of risk-reducing bilateral mastectomy should be considered.

The lifetime risk for thyroid cancer (usually follicular, rarely papillary) is approximately 35% [36]. Annual thyroid ultrasound from the time of diagnosis, including childhood, should be performed according to NCCN recommendations [6].

The risk for endometrial cancer may be close to 28% [36]. There are no data on screening for endometrial cancer. Routine transvaginal ultrasound has low sensitivity and specificity, especially in premenopausal women, whereas endometrial biopsy is highly sensitive and specific, but invasive. Therefore, screening with endometrial biopsy once in every 1–2 years may be considered, while hysterectomy should be discussed on a case-by-case basis, according to NCCN guidelines [6].

Half as many individuals with Cowden syndrome have adenomatous or hyperplastic colorectal polyps associated with early-onset (<50 years of age) colorectal cancer in 13% of patients [37]. Routine colonoscopy should be performed from the age of 35 once in every five years or more frequently, if the patient is symptomatic or polyps are found. However, screening should start 5–10 years before the age of the earliest case of colorectal cancer in the family.

Renal carcinoma may be present in up to 30% of patients. Melanoma skin cancer is also increased in patients with Cowden disease and may occur in 5% of patients [38]. Yearly to biennial renal imaging (preferably through CT or MRI) beginning at the age of 40 is recommended to screen renal cell carcinoma, while yearly dermatologic evaluation could help to detect early melanoma.

Brain tumors as well as vascular malformations occasionally affect individuals with Cowden syndrome. Cerebellar dysplastic gangliocytoma (Lhermitte-Duclos disease), a rare central nervous system tumor, can also be found in Cowden syndrome. However, the risk of developing these conditions is not well defined [39]. In the presence of neurological symptoms, especially in children, assessment of psychomotor abilities and brain MRI should be performed [6].

## 4. Lynch Syndrome

Lynch syndrome is caused by a germline mutation in one of four DNA mismatch repair genes (*MLH1*, *MSH2*, *MSH6*, or *PMS2*) [40] or deletions in the *EPCAM* gene resulting in *MSH2* silencing [41]. The estimated population frequency is 1 : 370 to 1 : 2,000 in Western populations [42]. Lynch syndrome is characterized by an increased lifetime risk for colorectal cancer (48–57% vs. 4.5%), endometrial cancer (43–57% vs. 2.7%), and other cancers including stomach (up to 13%), ovary (up to 24%), small bowel, hepatobiliary tract, urinary tract, brain, and skin [43].

Guidelines for cancer screening in patients with Lynch syndrome have been proposed by several groups including the American College of Gastroenterology, United States Multi-Society Task Force on Colorectal Cancer [44], European Hereditary Tumor Group [45], American Society of Clinical Oncology [46], and National Comprehensive Cancer Network [6].

Colonoscopy is recommended once in every 1–2 years starting from 20 to 25 years of age or 2–5 years before than the youngest diagnosis age in the family. Moreover, chromoendoscopy is a promising technique that could facilitate the detection of lesions and flat adenomas [47].

Regarding gynaecologic cancers, lifetime risk varies according to mutated gene and patient's age [48]. Transvaginal ultrasound and serum CA-125 testing were shown to be neither sufficiently sensitive nor specific to warrant a routine recommendation for early detection of endometrial and ovarian cancers. However, they may be required at clinicians' discretion in assessing tumor risk on a case-by-case basis [6]. Annual endometrial biopsy can be used as a screening tool for endometrial cancer because of its high sensitivity and sensibility [6]. Total hysterectomy is an option that may be considered to reduce the risk of endometrial cancer in women with Lynch syndrome; likewise, bilateral salpingo-oophorectomy may reduce the incidence of ovarian cancer [49]. Since there is no effective screening for gynaecologic cancers, women should be educated on relevant symptoms such as abnormal uterine bleeding, pelvic or abdominal pain, bloating, dyspepsia, or increased urinary frequency or urgency.

Regarding gastric, duodenal, and more distant small bowel cancer, there is insufficient evidence to recommend surveillance [50], except for individuals with relevant family history of these tumors [51]. Besides, esophagogastroduodenoscopy extended to the duodenum or into the jejunum once in every 3–5 years starting from 40 years of age should be considered in case of mutation in *MLH1*, *MSH2,* or *EPCAM* [45]. Considering that infection with *Helicobacter pylori* is a cause of gastric cancer, testing and treating for this bacterium is suggested [46].

There is no clear evidence to support screening for urinary tract cancer, except for individuals with a family history of urothelial cancer or *MSH2* mutation who may benefit from annual urinalysis beginning at 30–35 years of age [6]. The International Cancer of the Pancreas Screening (CAPS) Consortium recommends screening for pancreatic cancer in patients with Lynch syndrome and one first-degree relative with pancreatic cancer [52]. Nonetheless, no protocol for pancreatic cancer screening has been established yet. The NCCN panel therefore recommends MRI and endoscopic ultrasonography as screening modalities to be performed at high-volume centres with multidisciplinary teams and preferably in a research protocol [6]. By reason of the increased risk for brain cancer, in addition, annual physical and neurologic examination from 25 to 30 years of age may be considered, although no data support this practice [6].

Some studies have shown that mutations in *MLH1* and *MSH2*, and less frequently in *PMS2* and *MSH6*, could be associated with increased breast cancer risk [53–55]. Nevertheless, no specific recommendations for breast screening in women with Lynch syndrome have been made available so far, beyond those offered to the average risk population [6]. Finally, a study suggested an increased risk for prostate cancer in men with Lynch syndrome [56]. However, there is no sufficient evidence to recommend different prostate cancer screening from the rest of the population [6].

## 5. Diffuse Gastric Cancer Syndrome

Hereditary diffuse gastric cancer is a cancer susceptibility syndrome defined by the early onset of diffuse gastric cancer with or without lobular breast cancer. It is mainly caused by germline mutations in the epithelial cadherin (*CDH1*) gene. A most serious problem is that genetic diagnosis remains unknown in up to 60% of patients [57]. The risk for symptomatic gastric cancer, occurring by the age of 80, ranged between 67 and 70% in men and 56 and 83% in women, whereas the risk for breast cancer among women, especially the lobular phenotype, amounted to 52% [8].

Prophylactic total gastrectomy is strongly recommended between 18 and 40 years of age [58]. Screening by esophagogastroduodenoscopy with multiple random biopsy once in every 6–12 months should be reserved to patients who cannot undergo prophylactic total gastrectomy, since upper endoscopy may not detect early precursor lesions [59].

In women, finally, annual mammogram with consideration of breast MRI with contrast beginning at the age of 30 (or prior to that, with a family history of breast cancer before the age of 25) is recommended by NCCN guidelines [6]. However, given the high lifetime risk and the low sensitivity of mammography for lobular breast cancer, the added value of MRI over mammography seems high in this situation [60]. Risk-reducing mastectomy may be discussed with these carriers, depending on family history [6].

## 6. Peutz–Jeghers Syndrome

Germline pathogenic alterations in *STK11* are associated with Peutz–Jeghers syndrome. This is an autosomal dominant disorder characterized by hamartomatous gastrointestinal polyps, mucocutaneous pigmentation, and an increased risk of colorectal, gastric, pancreatic, gallbladder, small bowel, gynaecologic (uterus, cervix, and ovary), breast, testicular, and lung cancers [9].

Regarding the risk of colorectal, gastric, and small bowel cancers, colonoscopy, upper endoscopy, and capsule endoscopy should be recommended once in every 2–3 years, starting from the late teens [61]. Moreover, the American College of Gastroenterology recommends magnetic resonance cholangiopancreatography with contrast or endoscopic ultrasound once in every 1–2 years from 30 years of age, in order to detect early pancreatic cancer [62].

For breast cancer, annual mammogram and breast MRI screening with contrast should be recommended from 25 years of age. In women, transvaginal ultrasound, serum CA-125, and pelvic exam with Pap smear should be proposed annually beginning at 18 years of age. No data on the benefit of risk-reducing mastectomy are available, so that this procedure may be considered based on family history [6].

In males, annual testicular exam and, subsequently, ultrasound in case of symptomaticity or abnormality on exam are suggested from birth to the teen years [62].

## 7. Neurofibromatosis Type 1

Pathogenic variants of *NF1* cause neurofibromatosis type 1. This is an autosomal dominant HCS associated with increased risk for nervous system tumors (especially malignant peripheral nerve sheath tumors), gastrointestinal stromal tumors, and breast cancer [10].

The American Academy of Paediatrics and the American College of Medical Genetics and Genomics have published guidelines for children and adult surveillance [63, 64]. Annual physical examination, annual ophthalmologic examination in children (less frequently in adults), regular developmental assessment in children, regular blood pressure monitoring, and MRI for followup of clinically suspected intracranial tumors and other internal tumors are recommended. Additionally, annual mammography, possibly associated with breast MRI, is suggested between 30 and 50 years of age [65]. After 50 years of age, breast cancer risk in women with *NF1* mutation becomes similar to that of the rest of the population. Breast MRI could therefore be discontinued [66], while mammography can be performed at longer intervals. No data on the benefit of risk-reducing mastectomy are available, so that this procedure may be considered based on family history [6].

## 8. Other Breast and Ovarian Cancer Predisposition Genes

In addition to the known high-penetrance pathogenic variants of *BRCA1/2*, mutations in other intermediate or low-penetrant genes can increase the risk of breast and/or ovarian cancer. According to a retrospective analysis, these mutations account for 7.4% of patients who met the NCCN criteria for *BRCA1/2* mutation test [6]. The most common are *PALB2*, *ATM,* and *CHEK2* [67].

### 8.1. PALB2

It is estimated that 0.6%–3% of patients with breast cancer harbour a mutation in *PALB2* (partner and localizer of *BRCA2*) [11, 15, 68]. Moreover, women carrying pathogenetic variants of *PALB2* have a 35% lifetime risk to develop breast cancer by 70 years of age. The higher the number of relatives affected, the higher the risk [69]. Breast ultrasound and MRI are recommended yearly from 25 to 29 years of age, alternating once in every six months. Annual mammogram and breast MRI screening are alternatively recommended once in every six months, starting at 30 until 65 years of age [6].

Some studies highlight a possible association between *PALB2* mutations and ovarian cancer. Recently, *PALB2* has also been reported to be a new pancreatic cancer susceptibility gene [70]. However, the associated risks are unclear and not well-estimated. Furthermore, no effective screening method is available for ovarian or pancreatic cancer. Screening and/or risk-reducing surgery should be individualized based on familial history [71].

### 8.2. ATM, CHEK2, NBN, and BARD1

Individuals carrying heterozygous pathogenic variants in *ATM* have a 33% cumulative lifetime risk for breast cancer by 80 years of age [12]. Mammogram with consideration of breast MRI is recommended yearly from 40 years of age [6]. No data are available on the benefit of risk-reducing mastectomy, so that this procedure may be considered based on family history [6]. *ATM* heterozygous pathogenic variants have been reported in some cases of familial ovarian [15], pancreatic [72], and prostate [73] cancer. Screening for pancreatic and ovarian cancers in carriers of *ATM* pathogenic variants is not recommended in the absence of familial antecedents, while men should be encouraged to participate in prostate cancer screening [6]. Homozygous or compound heterozygous *ATM* mutations cause ataxia telangiectasia, a syndrome characterized by progressive cerebellar ataxia, oculomotor apraxia, immunodeficiency, and general increased risk of malignancies [74].

The rate of *CHEK2* germline mutation is higher in Northern European countries than in Mediterranean ones. Certain mutations in the *CHEK2* gene (c.1100delC and I157T) are associated with increased breast cancer risk, with a cumulative lifetime risk ranging from 28% to 37% depending on family history [13, 75]. Mammogram and breast MRI once a year start at 40 years of age [6]. No data are available on the benefit of risk-reducing mastectomy, so that this procedure may be considered based on family history [6]. Within families carrying pathogenic *CHEK2* variants, there is also an increased risk of other malignancies including colon, prostate, kidney, bladder, and thyroid cancers [76], with the vast majority of data for the c1100delC variant. Colonoscopy once in every five years, beginning from 40 years of age or 10 years earlier than the age of diagnosis for any first-degree relative with colorectal cancer, is recommended in individuals carrying *CHEK2* mutations [6]. Currently, there are no specific medical management guidelines to address the possible risk of developing prostate, kidney, bladder, and thyroid cancer in these individuals.

Individuals with slavic founder heterozygous *NBN* mutation 675del5 have an increased risk of developing numerous types of cancer, including breast (up to 30% at 80 years of age) and ovarian cancer. Moreover, an unestimated increased risk of prostate cancer at 80 years of age is also apparent in men [77]. The presence of biallelic hypomorphic *NBN* mutations leads to the Nijmegen breakage syndrome, a rare autosomal recessive syndrome of chromosomal instability mainly characterized by microcephaly at birth, combined immunodeficiency, and predisposition to malignancies. Approximately 40% of the affected patients develop a malignancy before the age of 21 [14]. In slavic mutation carriers, breast MRI is recommended yearly from 40 years of age, whereas no recommendations are provided for ovarian and prostate cancer screening [6]. No data are available on the benefit of risk-reducing mastectomy, so that this procedure may be considered based on family history [6].

Deleterious *BARD1* germline variants are significantly associated with early-onset breast cancer, according to recent studies [78, 79]. On the grounds of these data, intensified breast cancer screening programs should be offered to women carrying pathogenic *BARD1* gene variants. However, the starting age and the frequency of mammogram and/or breast MRI have not been established yet.

### 8.3. BRIP1, RAD51C, and RAD51D

Mutations in *BRIP1*, *RAD51C,* or *RAD51D* are associated with an increased risk of developing ovarian cancer. The prevalence rate of *BRIP1*, *RAD51C,* or *RAD51D* pathogenic variants is about 1% in women with ovarian cancer [15]. Nevertheless, there are no data supporting screening for ovarian cancer. Transvaginal ultrasound and serum CA-125 testing have not been shown to be sufficiently sensitive or specific, even in the setting of women at high risk of ovarian cancer due to an inherited mutation [6]. NCCN guidelines recommend that risk-reducing salpingo-oophorectomy should be considered beginning at 45–50 years of age [6]. At present, *BRIP1*, *RAD51C,* or *RAD51D* are not associated with an increased risk for breast cancer [7].

## 9. Conclusions

In the last years, a large number of large case-control studies have shown the correlation between mutations in some genes and an increased risk of developing breast and/or ovarian cancer, explaining tumor recurrence in those families where mutations in *BRCA1/2* were not found. However, the lifetime risk in case of low-penetrant genes has not been defined yet and additional prospective studies are needed to establish a more customised screening program for carriers. Summary of the recommendations for each predisposition gene discussed in the present review is reported in Table 1.

## Figures and Tables

**Table 1:** Summary of the recommendations for each predisposition gene.

Predisposition genes	Cancer risk	Lifetime risk	Surveillance
*High-penetrance genes for breast and/or ovarian cancer*			

TP53	Adrenocortical gland	6–13% [25]	Ultrasound of abdomen and pelvis: every 3–4 mos, birth to age 18 yrs [27]
	Breast	54% [25]	Clinical breast examination: every 6–12 mos, age ≥ 20 yrs
			Breast MRI screening with contrast (with or without mammogram): annually, age 20–75 yrs [27]^*∗*^
	Central nervous system	6–19% [25]	Neurologic exam: annually, all ages
			Brain MRI: annually [27]
	Sarcomas	5–22% [25]	Whole-body MRI: annually, all ages
			Ultrasound of abdomen and pelvis: annually, age ≥18 yrs [27]
	Hematologic tumors	NA	Periodic blood test if increased risk for myelodysplastic syndrome or leukaemia [28]
	Gastrointestinal system	NA	Upper endoscopy and colonoscopy: every 2–5 yrs, age ≥25 yrs [27]
	Skin	NA	Dermatologic exam: annually, age ≥18 yrs [27]

PTEN	Breast	85% [5]	Clinical breast examination: every 6 mos, age ≥ 25 yrs
			Mammogram and breast MRI with contrast: annually, age 30–75 yrs [6]^*∗*^
	Thyroid	35% [36]	Ultrasound of thyroid: annually, all ages [6]
	Endometrium	28% [36]	Endometrial biopsy: every 1–2 yrs [6]^*∗*^
	Colon and rectum	9% [36]	Colonoscopy: every 5 yrs, age ≥ 35 yrs [6]
	Kidney	30% [36]	CT or MRI of abdomen: every 1–2 yrs, age ≥ 40 yrs [6]
	Melanoma	5% [38]	Dermatologic exam: annually, age ≥18 yrs [38]

CDH1	Stomach	56–83% [8]	Upper endoscopy: every 6–12 mos, age ≥ 18 yrs [59]^*∗*^
	Breast	52% [8]	Mammogram and breast MRI with contrast: annually, age ≥ 30 yrs [6]^*∗*^

STK11	Colon and rectum	39% [9]	Colonoscopy: every 2–3 yrs, age ≥ 18 yrs [61]
	Stomach	29% [9]	Upper endoscopy: every 2–3 yrs, age ≥ 18 yrs [61]
	Small bowel	13% [9]	Capsule endoscopy: every 2–3 yrs, age ≥ 18 yrs [61]
	Pancreas	11–36% [9]	MR cholangiopancreatography with contrast or endoscopic ultrasound: every 1–2 yrs, age ≥ 30 yrs [62]
	Breast	32–54% [9]	Clinical breast examination: every 6 mos, age ≥ 20 yrs
			Mammogram and breast MRI with contrast: annually, age ≥ 25 yrs [6]^*∗*^
	Ovary, cervix, and uterus	9–21% [9]	Transvaginal ultrasound, serum CA 125, pelvic exam with pap smear: annually, age ≥ 18 yrs [6]
	Testis	9% [9]	Testicular exam: annually, until 18 yrs [62]
	Lung	7–17% [9]	Not recommended
*Low-/moderate-penetrance genes for breast and/or ovarian cancer*			

PALB2	Breast	35% [69]	Mammogram and breast MRI with contrast: annually, age ≥ 30 yrs [6]^*∗*^
	Ovary, pancreas	NA	Not recommended

CHEK2	Breast	28–37% [13, 75]	Mammogram and breast MRI with contrast: annually, age ≥ 40 yrs [6]^*∗*^
	Colon	NA	Colonoscopy: every 5 yrs, age ≥ 40 yrs [6]
	Prostate, kidney, bladder, and thyroid	NA	Not recommended

NBN (*675del5*)	Breast	Up to 30% [77]	Breast MRI with contrast: annually, age ≥ 40 yrs [6]^*∗*^
	Ovary and prostate	NA	Not recommended

MLH1, MSH2, MSH6, PMS2, EPCAM	Colon and rectum	48–57% [43]	Colonoscopy: every 1-2 yrs, age ≥ 20–25 yrs [6]
	Endometrium	43–57% [43]	Not recommended^*∗*^
	Ovary	Up to 24% [43]	Not recommended^*∗*^
	Stomach, small bowel	4–13% [43]	Upper endoscopy: every 3–5 yrs, age ≥ 40 yrs if relevant family history or mutation in *MLH1*, *MSH2* or *EPCAM* [45, 51]
	Hepatobiliary tract	Up to 4% [43]	In research protocol [6]
	Urinary tract	Up to 25% [43]	Urinalysis: annually, age ≥ 30–35 yrs if relevant family history or *MSH2* mutation [6]
	Brain	1–4% [43]	Physical and neurologic examination: annually, age ≥ 25–30 yrs [6]
	Breast (*MSH2*, *MLH1*, *PMS2,* or *MSH6*)	11–18% [53–55]	Not recommended
	Prostate	NA	Not recommended

ATM	Breast	33% [12]	Mammogram with consideration of breast MRI with contrast: annually, age ≥ 40 yrs [6]^*∗*^
	Ovary, prostate, and pancreas	NA	Not recommended
BRIP1, RAD51C, RAD51D	Ovary	Up to 10% [15]	Not recommended^*∗*^

NF1	Nervous system	8–16% [64]	Physical and eye examination: annually, every age [63, 64]
	Breast	17% [64]	Mammogram and breast MRI with contrast: annually, age 30–50 yrs [65]^*∗*^
BARD1	Breast	NA	Not recommended

NA = not available, ^*∗*^Risk-reducing surgery can be considered based on type of mutation and family history.

## References

[1] Garber J. E., Offit K. (2005). Hereditary cancer predisposition syndromes. *Journal of Clinical Oncology*.

[2] Hall J., Lee M., Newman B. (1990). Linkage of early-onset familial breast cancer to chromosome 17q21. *Science*.

[3] Wooster R., Neuhausen S., Mangion J. (1994). Localization of a breast cancer susceptibility gene, BRCA2, to chromosome 13q12-13. *Science*.

[4] Schneider K., Zelley K., Nichols K. E., Adam M. P., Ardinger H. H., Pagon R. A. (1999). Li-fraumeni syndrome. *GeneReviews® [Internet]*.

[5] Eng C., Adam M. P., Ardinger H. H., Pagon R. A. (2001). PTEN hamartoma tumor syndrome. *GeneReviews® [Internet]*.

[6] National Comprehensive Cancer Network (2019). Genetic/familial high-risk assessment: colorectal. *NCCN Clinical Practice Guidelines in Oncology (NCCN Guidelines) 2019*.

[7] Suszynska M., Klonowska K., Jasinska A. J., Kozlowski P. (2019). Large-scale meta-analysis of mutations identified in panels of breast/ovarian cancer-related genes - providing evidence of cancer predisposition genes. *Gynecologic Oncology*.

[8] Kaurah P., MacMillan A., Boyd N. (2007). Founder and recurrent CDH1 mutations in families with hereditary diffuse gastric cancer. *JAMA*.

[9] McGarrity T. J., Amos C. I., Baker M. J., Adam M. P., Ardinger H. H., Pagon R. A. (2001). Peutz-jeghers syndrome. *GeneReviews® [Internet]*.

[10] Friedman J. M., Adam M. P., Ardinger H. H., Pagon R. A. (1998). Neurofibromatosis 1. *GeneReviews® [Internet]*.

[11] Couch F. J., Shimelis H., Hu C. (2017). Associations between cancer predisposition testing panel genes and breast cancer. *JAMA Oncology*.

[12] Marabelli M., Cheng S.-C., Parmigiani G. (2016). Penetrance ofATMGene mutations in breast cancer: a meta-analysis of different measures of risk. *Genetic Epidemiology*.

[13] Cybulski C., Wokołorczyk D., Jakubowska A. (2011). Risk of breast cancer in women with a CHEK2 mutation with and without a family history of breast cancer. *Journal of Clinical Oncology*.

[14] Chrzanowska K. H., Gregorek H., Dembowska-Bagińska B., Kalina M. A., Digweed M. (2012). Nijmegen breakage syndrome (NBS). *Orphanet Journal of Rare Diseases*.

[15] Lu H.-M., Li S., Black M. H. (2019). Association of breast and ovarian cancers with predisposition genes identified by large-scale sequencing. *JAMA Oncology*.

[16] Toss A., Venturelli M., Molinaro E. (2019). Hereditary pancreatic cancer: a retrospective single-center study of 5143 italian families with history of BRCA-related malignancies. *Cancers*.

[17] Grandi G., Sammarini M., Chiara Del Savio M., Toss A., Facchinetti F. (2019). Combined hormonal contraceptives in BRCA gene mutation carriers: why not?. *The European Journal of Contraception & Reproductive Health Care*.

[18] Cortesi L., Canossi B., Battista R. (2019). Breast ultrasonography (BU) in the screening protocol for women at hereditary-familial risk of breast cancer: has the time come to rethink the role of BU according to different risk categories?. *International Journal of Cancer*.

[19] Cortesi L., De Matteis E., Toss A. (2017). Evaluation of transvaginal ultrasound plus CA-125 measurement and prophylactic salpingo-oophorectomy in women at different risk levels of ovarian cancer: the modena study group cohort study. *Oncology*.

[20] Toss A., Grandi G., Cagnacci A. (2017). The impact of reproductive life on breast cancer risk in women with family history or BRCA mutation. *Oncotarget*.

[21] Razzaboni E., Toss A., Cortesi L. (2013). Acceptability and adherence in a chemoprevention trial among women at increased risk for breast cancer attending the modena familial breast and ovarian cancer center (Italy). *The Breast Journal*.

[22] Patel V. L., Busch E. L., Friebel T. M. (2020). Association of genomic domains in BRCA1 and BRCA2 with prostate cancer risk and aggressiveness. *Cancer Research*.

[23] Nielsen S. M., Eccles D. M., Romero I. L (2018). Genetic testing and clinical management practices for variants in non-BRCA1/2 breast (and breast/ovarian) cancer susceptibility genes: an international survey by the evidence-based Network for the interpretation of germline mutant alleles (ENIGMA) clinical working group. *JCO Precision Oncology*.

[24] Kamihara J., Schneider K. (2014). Li-Fraumeni syndrome. https://www.orpha.net/consor/cgi-bin/Disease_Search.php?lng=EN&data_id=196.

[25] Mai P. L., Best A. F., Peters J. A. (2016). Risks of first and subsequent cancers amongTP53mutation carriers in the National Cancer Institute Li-Fraumeni syndrome cohort. *Cancer*.

[26] Guha T., Malkin D., Inherited “ (2017). TP53 mutations and the Li-Fraumeni syndrome. *Cold Spring Harb Perspect Med*.

[27] Villani A., Shore A., Wasserman J. D. (2016). Biochemical and imaging surveillance in germline TP53 mutation carriers with Li-Fraumeni syndrome: 11 year follow-up of a prospective observational study. *The Lancet Oncology*.

[28] Kratz C. P., Achatz M. I., Brugières L. (2017). Cancer screening recommendations for individuals with Li-Fraumeni syndrome. *Clinical Cancer Research*.

[29] Gondim G. R. M., Formiga M. N. C., Castro D. G. (2018). Adjuvant radiation therapy in patients with breast cancer and Li-Fraumeni syndrome: oncologic results and incidence of second neoplasms. *Journal of Clinical Oncology*.

[30] Heymann S., Delaloge S., Rahal A. (2010). Radio-induced malignancies after breast cancer postoperative radiotherapy in patients with Li-Fraumeni syndrome. *Radiation Oncology*.

[31] Ballinger M. L., Best A., Mai P. L. (2017). Baseline surveillance in Li-Fraumeni syndrome using whole-body magnetic resonance imaging. *JAMA Oncology*.

[32] Villani A., Tabori U., Schiffman J. (2011). Biochemical and imaging surveillance in germline TP53 mutation carriers with Li-Fraumeni syndrome: a prospective observational study. *The Lancet Oncology*.

[33] Pilarski R. (2009). Cowden syndrome: a critical review of the clinical literature. *Journal of Genetic Counseling*.

[34] Pilarski R., Eng C. (2004). Will the real Cowden syndrome please stand up (again)? Expanding mutational and clinical spectra of the PTEN hamartoma tumour syndrome. *Journal of Medical Genetics*.

[35] Hobert J. A., Eng C. (2009). PTEN hamartoma tumor syndrome: an overview. *Genetics in Medicine*.

[36] Tan M.-H., Mester J. L., Ngeow J., Rybicki L. A., Orloff M. S., Eng C. (2012). Lifetime cancer risks in individuals with germline PTEN mutations. *Clinical Cancer Research*.

[37] Heald B., Mester J., Rybicki L., Orloff M. S., Burke C. A., Eng C. (2010). Frequent gastrointestinal polyps and colorectal adenocarcinomas in a prospective series of PTEN mutation carriers. *Gastroenterology*.

[38] Garofola C., Gross G. P. (2020). Cowden disease (multiple hamartoma syndrome). *StatPearls [Internet]*.

[39] Pilarski R., Burt R., Kohlman W., Pho L., Shannon K. M., Swisher E. (2013). Cowden syndrome and the PTEN hamartoma tumor syndrome: systematic review and revised diagnostic criteria. *JNCI Journal of the National Cancer Institute*.

[40] Boland C. R., Goel A. (2010). Microsatellite instability in colorectal cancer. *Gastroenterology*.

[41] Kempers M. J., Kuiper R. P., Ockeloen C. W. (2011). Risk of colorectal and endometrial cancers in EPCAM deletion-positive Lynch syndrome: a cohort study. *The Lancet Oncology*.

[42] Hampel H., de la Chapelle A. (2013). How do we approach the goal of identifying everybody with Lynch Syndrome?. *Familial Cancer*.

[43] Kohlmann W., Gruber S. B., Adam M. P., Ardinger H. H., Pagon R. A. (2004). Lynch syndrome. *GeneReviews® [Internet]*.

[44] Giardiello F. M., Allen J. I., Axilbund J. E. (2014). Guidelines on genetic evaluation and management of Lynch syndrome: a consensus statement by the US multi-society Task Force on colorectal cancer. *American Journal of Gastroenterology*.

[45] Vasen H. F. A., Blanco I., Aktan-Collan K. (2013). Revised guidelines for the clinical management of Lynch syndrome (HNPCC): recommendations by a group of European experts. *Gut*.

[46] Stoffel E. M., Mangu P. B., Gruber S. B. (2015). Hereditary colorectal cancer syndromes: American society of clinical Oncology clinical practice guideline endorsement of the familial risk-colorectal cancer: European society for medical Oncology clinical practice guidelines. *Journal of Clinical Oncology*.

[47] Haanstra J. F., Kleibeuker J. H., Koornstra J. J. (2013). Role of new endoscopic techniques in Lynch syndrome. *Familial Cancer*.

[48] Møller P., Seppälä T., Bernstein I. (2017). Cancer incidence and survival in Lynch syndrome patients receiving colonoscopic and gynaecological surveillance: first report from the prospective Lynch syndrome database. *Gut*.

[49] Schmeler K. M., Lynch H. T., Chen L.-m. (2006). Prophylactic surgery to reduce the risk of gynecologic cancers in the Lynch syndrome. *New England Journal of Medicine*.

[50] Renkonen-Sinisalo L., Sipponen P., Aarnio M. (2002). No support for endoscopic surveillance for gastric cancer in hereditary non-polyposis colorectal cancer. *Scandinavian Journal of Gastroenterology*.

[51] Clinical Kim J., Braun D., Ukaegbu C. (2019). Factors associated with gastric cancer in individuals with Lynch syndrome. *Clinical Gastroenterology and Hepatology*.

[52] Canto M. I., Harinck F., Hruban R. H. (2013). International Cancer of the Pancreas Screening (CAPS) Consortium summit on the management of patients with increased risk for familial pancreatic cancer. *Gut*.

[53] Harkness E. F., Barrow E., Newton K. (2015). Lynch syndrome caused byMLH1mutations is associated with an increased risk of breast cancer: a cohort study. *Journal of Medical Genetics*.

[54] Goldberg M., Bell K., Aronson M. (2017). Association between the Lynch syndrome gene MSH2 and breast cancer susceptibility in a Canadian familial cancer registry. *Journal of Medical Genetics*.

[55] Roberts M. E., Jackson S. A., Susswein L. R. (2018). MSH6 and PMS2 germ-line pathogenic variants implicated in Lynch syndrome are associated with breast cancer. *Genetics in Medicine*.

[56] Haraldsdottir S., Hampel H., Wei L. (2014). Prostate cancer incidence in males with Lynch syndrome. *Genetics in Medicine*.

[57] Tan R. Y. C., Ngeow J. (2015). Hereditary diffuse gastric cancer: what the clinician should know. *World Journal of Gastrointestinal Oncology*.

[58] Seevaratnam R., Coburn N., Cardoso R. (2012). A systematic review of the indications for genetic testing and prophylactic gastrectomy among patients with hereditary diffuse gastric cancer. *Gastric Cancer*.

[59] Lim Y. C., di Pietro M., O’Donovan M. (2014). Prospective cohort study assessing outcomes of patients from families fulfilling criteria for hereditary diffuse gastric cancer undergoing endoscopic surveillance. *Gastrointestinal Endoscopy*.

[60] Johnson K., Sarma D., Hwang E. S. (2015). Lobular breast cancer series: imaging. *Breast Cancer Research*.

[61] Dunlop M. G. (2002). Guidance on gastrointestinal surveillance for hereditary non-polyposis colorectal cancer, familial adenomatous polypolis, juvenile polyposis, and Peutz-Jeghers syndrome. *Gut*.

[62] Syngal S., Brand R. E., Church J. M., Giardiello F. M., Hampel H. L., Burt R. W. (2015). ACG clinical guideline: genetic testing and management of hereditary gastrointestinal cancer syndromes. *American Journal of Gastroenterology*.

[63] Miller D. T., Freedenberg D., Schorry E. (2019). Health supervision for children with neurofibromatosis type 1. *Pediatrics*.

[64] Stewart D. R., Korf B. R., Nathanson K. L., Stevenson D. A., Yohay K. (2018). Care of adults with neurofibromatosis type 1: a clinical practice resource of the American College of Medical Genetics and Genomics (ACMG). *Genetics in Medicine*.

[65] Evans D. G. (2012). Are we ready for targeted early breast cancer detection strategies in women with NF1 aged 30-49 years?. *American Journal of Medical Genetics Part A*.

[66] Seminog O. O., Goldacre M. J. (2015). Age-specific risk of breast cancer in women with neurofibromatosis type 1. *British Journal of Cancer*.

[67] Kurian A. W., Hughes E., Handorf E. A. (2017). Breast and ovarian cancer penetrance estimates derived from germline multiple-gene sequencing results in women. *JCO Precision Oncology*.

[68] Casadei S., Norquist B. M., Walsh T. (2011). Contribution of inherited mutations in the BRCA2-interacting protein PALB2 to familial breast cancer. *Cancer Research*.

[69] Antoniou A. C., Casadei S., Heikkinen T. (2014). Breast-cancer risk in families with mutations in PALB2. *New England Journal of Medicine*.

[70] Jones S., Hruban R. H., Kamiyama M. (2009). Exomic sequencing identifies PALB2 as a pancreatic cancer susceptibility gene. *Science*.

[71] Tung N., Domchek S. M., Stadler Z. (2016). Counselling framework for moderate-penetrance cancer-susceptibility mutations. *Nature Reviews Clinical Oncology*.

[72] Shindo K., Yu J., Suenaga M. (2017). Deleterious germline mutations in patients with apparently sporadic pancreatic adenocarcinoma. *Journal of Clinical Oncology*.

[73] Pilié P. G., Johnson A. M., Hanson K. L. (2017). Germline genetic variants in men with prostate cancer and one or more additional cancers. *Cancer*.

[74] Chun H. H., Gatti A. R. (2004). Ataxia-teleangiectasia, an evolving phenotype. *DNA Repair*.

[75] Weischer M., Bojesen S. E., Ellervik C., Tybjærg-Hansen A., Nordestgaard B. G. (2008). CHEK2^∗^1100delC genotyping for clinical assessment of breast cancer risk: meta-analyses of 26,000 patient cases and 27,000 controls. *Journal of Clinical Oncology*.

[76] Cybulski C., Górski B., Huzarski T. (2004). CHEK2 is a multiorgan cancer susceptibility gene. *The American Journal of Human Genetics*.

[77] Seemanová E., Jarolim P., Seeman P. (2007). Cancer risk of heterozygotes with the NBN founder mutation. *Journal of the National Cancer Institute*.

[78] Weber-Lassalle N., Borde J., Weber-Lassalle K. (2019). Germline loss-of-function variants in the BARD1 gene are associated with early-onset familial breast cancer but not ovarian cancer. *Breast Cancer Res*.

[79] Suszynska M., Kluzniak W., Wokolorczyk D. (2019). BARD1 is A Low/Moderate breast cancer risk gene: evidence based on an association study of the central European p.Q564X recurrent mutation. *Cancers*.

